# Supplementary material for: Are carbon emissions trading and green financial instruments synergistic? -Comprehensive quantitative research based on content analysis
Source: PLoS One. 2024 Mar 7;19(3):e0298601. doi: 10.1371/journal.pone.0298601 (PMC10919601; doi:10.1371/journal.pone.0298601)
Supplement: S1 Data — (ZIP) [file pone.0298601.s001.zip › Data/地级市Z_F工作报告.zip!/╣π╢1⁄2/╔╟╬▓╩╨/└┤╫╘╣1⁄2╓┌║┼í╛┬φ┐╦╩2╛▌═°í┐_2015.docx]

各位代表：

　　我代表市人民政府向大会作政府工作报告，请予审议，并请政协各位委员和其他列席人员提出意见。

2014年工作回顾

　　2014年，在省委、省政府及市委的正确领导下，在市人大及其常委会和市政协的监督支持下，市政府全面贯彻落实党的十八大、十八届三中、四中全会和省委十一届三次全会精神及省《关于进一步促进粤东西北地区振兴发展的决定》，心无旁骛向西发展，主动融入珠三角，全面对接深圳帮扶，围绕“一个目标”,守住“两条底线”,狠抓“三大抓手”，开展“四大行动”，确保经济发展稳中有进，社会保持和谐稳定。全市完成生产总值716.99亿元，增长8.9%。

　　（一）产业优化升级加快

　　重点项目进展顺利。固定资产投资500.97亿元，增长16.5%。11项省重点项目完成投资108亿元，完成年度计划100.5%；48项市重点项目完成投资128亿元，完成年度计划96%。华润海丰电厂2号和1号机组预计今年初和上半年建成投产；陆丰核电“两评”报告获国家批准，项目审批取得关键性进展；陆丰宝丽华甲湖湾电厂核准已在公示中，有望近期正式动工；腾讯云计算中心完成设备安装。

　　工业经济持续发力。规模以上工业增加值261.91亿元，增长14.4%。电子信息、电力能源、工艺品制造、纺织服装四大主导产业实现增加值178.96亿元，增长12.7%，占规上工业增加值68.3%。先进制造业增加值73.88亿元，增长18.6%；高新技术制造业增加值58.59亿元，增长15.3%。信利投入7.59亿元新添摄像模组和菲林触摸屏2条生产线，新增产值44亿元。科技创新能力不断增强，新增研发机构3家、企业研发中心2家，高新技术企业4家；专利申请595件，专利授权458件；新增省级专业镇2家。

　　“三农”工作扎实推进。农业增加值114.14亿元，增长4.1%。粮食总产量44.88万吨，增长7.6%。农村常住居民人均可支配收入10415元，增长9.9%。海亮、三禾、协兴、骏丰农产品、共拓实业等一批现代农业产业项目顺利落地。全市累计流转土地13.2万亩，比上年增加1.2万亩。建成高标准基本农田18.98万亩，农业机械化综合水平达52%。大中型水库移民安居工程建设100%完成。陆河县被授予“国家有机产品认证示范创建县”称号。农村集体资产资源交易平台建设和陆河土地承包经营权确权登记颁证试点稳步开展。

　　商贸旅游日趋繁荣。社会消费品零售总额520.11亿元，增长9.8%。“万村千乡市场工程”顺利推进，完成236家“农家店”信息化改造。制订电子商务平台建设促进办法，开展与阿里巴巴等国内外大型电商平台合作。成功举办汕尾海洋文化旅游节、海丰可塘珠宝文化节、陆河青梅节等节庆活动，凤山祖庙旅游区被评为国家4A级旅游景区。全年接待过夜游客646.62万人次，实现旅游总收入96.44亿元，分别增长10.5%、17.6%。

　　（二）振兴发展成效明显

　　新区建设开局良好。新区专项规划抓紧修编，三个起步区控制性详规基本完成。全省首批、粤东首家股权基金9.3亿元落户我市，省建行批准新区基金融资60亿元，先行贷款5.8亿元。火车站站前广场及周边配套道路、环湖路东段、新湖大道、外环快速路首期工程加快推进，工业大道西段市政综合工程完成勘察设计招投标和环评可研。完成火车站片区一、二期和金町湾一期等征地工作，收储土地4500多亩。保利旅游综合地产、广物汽车城等项目开工建设。

　　产业园区建设全面提速。全市“1+5”产业园区共投入基础设施建设资金7.79亿元，征地13.44平方公里，动工项目63宗，计划总投资559.4亿元。深汕特别合作区发展规划获省批准，投资控股公司正式成立，汕尾市、海丰县已授权深汕特别合作区31项审批管理权限，华润新一代数据中心、晟火科技园、康强新材料等37个产业项目开工建设。汕尾高新技术开发区获省认定，海丰、陆丰、陆河、红海湾等产业园区建设进展顺利，广晟南储冷链、维业科技等一批项目落地建设。

　　交通基础设施建设加快。完成交通建设投资32.2亿元，比上年翻一番；其中公路建设投资27.2亿元，增长91.7%，新（改）建公路里程241公里。潮惠高速公路汕尾段全线建设有序推进，深汕高速公路长沙湾全互通立交工程动工建设，潭西互通立交工程前期工作抓紧推进。国道324线海丰县城段及梅陇穿城段加快建设,陆丰穿城段获省批复。华润海丰电厂5万吨级煤码头建成投入使用。广州至汕尾铁路、兴宁至汕尾高速公路、陆丰甲湖湾电厂10万吨级煤码头等项目前期工作加快推进。

　　（三）开放合作不断拓展

　　招商引资态势良好。实际利用外商直接投资1.63亿美元，增长6.8%。与深圳联合举办4次大型招商推介活动，参加广东21世纪海上丝绸之路国际博览会等招商活动，累计签约项目148宗。清华同方、深圳特建发集团、盐田港集团等一批企业落户我市。

　　外向经济动力增强。高新技术产品出口占汕头关区5市总量的57%，加工贸易“委托设计+自主品牌”生产方式出口比重达68%。汕尾港口岸红海湾港区通过国家验收。民营企业“走出去”步伐加快，国泰公司投资文莱最大海水养殖项目。

　　向西“融珠”开局良好。正式加入深莞惠经济圈，首次参加深莞惠经济圈（3+2）党政主要领导联席会议，在交通运输、产业合作、生态环保等领域，深化与深莞惠河四市共建合作。创新深汕两市结对帮扶机制，开展全方位、多层次的帮扶与合作。明确深汕两市结对区县各共建一个产业园区，切实增强帮扶实效。深圳180多名干部到我市开展对口帮扶工作，累计投入帮扶资金8.48亿元。

　　（四）深化改革稳步推进

　　重点改革任务顺利推进。认真落实中央和省深化改革举措，部署293项具体改革任务和45项2014年重点改革任务。供销合作社、医疗结算管理平台、劳动保障卡个人数据库等12项中央、省改革试点工作任务稳步实施。市发改、商务、城市综合执法等市直部门完成改革整合。市取消行政审批事项24项，下放和委托17项，调整和整合39项。开展“先照后证”商事登记制度改革，全年新登记各类企业和注册资本分别增长73.2%和302.3%。

　　投融资改革成效显著。我市首家改制地方银行汕尾农商行挂牌营业。广州万联证券、生命人寿保险公司进驻我市。海丰县申请发行7.5亿元企业债券取得重大进展。遮浪渔港首期工程等4个项目吸纳民间投资2.23亿元。完成市南告水电厂、汽运公司和中旅社等企业改制工作。信利光电上市进入辅导期。维明科技在广州股权交易中心挂牌交易。

　　其他领域改革稳步推进。市人民医院等市直公立医院改革正式实施，57个基层卫生医疗机构全面实施国家基本药物制度。网上办事大厅提前开展企业专属网页和市民个人网页建设。市、县两级土地使用权网上交易系统不断完善，市公共资源交易体制改革顺利完成。

　　（五）生态和谐社会稳定

　　生态文明建设加快。新一轮绿化行动深入推进，完成造林22.8万亩，建成生态景观林带72.8公里，新增森林公园8个、湿地公园3个，建成绿化美化示范点223个，全市森林覆盖率53.5%，比上年提高1.4个百分点。深入开展节能减排和污染治理，陆丰甲子、碣石污水处理厂项目加快建设，公平水库、螺河整治取得成效，淘汰黄标车、老旧车3128辆，完成省下达任务111.4%。市区空气质量指数达标天数为337天。

　　宜居城乡建设统筹推进。修编完善汕尾市城镇体系规划、城市总体规划，加快完善品清湖区域空间发展规划等重点片区规划。启动省文明城市和卫生城市创建工作。深入开展市区道路交通安全管理综合整治活动，大力整治市区占道经营和乱搭建，城市面貌有所改观。创建市级宜居城镇13个、宜居村庄29个，红海湾遮浪街道等4个镇（街）被评为省宜居示范城镇，城区马宫街道长沙村等10个村被评为省宜居示范村庄。陆河县生活垃圾无害化处理填埋场投入试运行，市生活垃圾无害化处理中心首期工程加紧建设，预计今年上半年可试运营。建成镇垃圾转运站50个、自然村垃圾收集点3321个，创建省卫生村119个,农村生活垃圾处理设施实现“一县一场”、“一镇一站”、“一村一点”。

　　社会管理不断加强。深入推进平安汕尾建设，以陆丰涉毒整治为重点，制订《关于加强禁毒工作的实施意见》，持续加大对制贩毒打击整治力度，全年侦破毒品案件778宗，抓获涉毒犯罪嫌疑人917人，缴获冰毒3495.32千克。开展涉枪、涉黄赌等“六大专项”行动，治安形势保持稳定。扎实做好信访维稳工作，省、市两级督办的重大矛盾纠纷化解率100%。村级“两委”100%顺利完成换届工作。计生、殡改、产品质量、食品药品安全、海防打私等管理工作得到加强。安全生产形势总体稳定。应急管理水平得到提高，陆丰核电项目公众沟通工作圆满完成,获省充分肯定。

　　（六）民生福祉逐步改善

　　十件民生实事稳步推进。全市投入民生事业93.34亿元，占公共财政预算支出的74.78%，解决了就业、教育、住房等一批民生热点难点问题。山区和农村边远地区义务教育学校教师岗位津贴补助标准提高到人均700元/月。为6万名农村妇女免费实施“两癌”筛查。全市保障性安居工程新开工建设1318套（户），基本建成1296套（户）。市人民群众来访接待厅等设施建设加快推进。完成农村公路硬底化187公里建设任务。

　　社会保障能力不断增强。城镇新增就业4.65万人，城镇登记失业率2.39%，农村劳动力转移就业率76.86%。参加城镇职工基本养老保险人数50.43万人，城乡居民养老保险人数92.4万人，城乡基本医疗保险300.5万人，全面完成年度任务。城乡居民基础养老金标准提高到每月80元，基本医疗保险补助标准提高到每人年320元。城乡居民低保补差标准分别提高到每人月335元和150元，纳入低保救助的生活困难群众10.3万人，发放低保金2.24亿元。

　　教育文化、扶贫开发等各项事业有新进步。教育创强取得新成果，建成省级教育强镇17个；率先在粤东西北地区实现公办中小学课室多媒体电教平台全覆盖，实现优质数字教育资源“班班通”。市特殊教育学校新校区建成使用。成功举办广东省（汕尾）“马思聪杯”小提琴邀请赛、中国龙舟公开赛（汕尾海丰站）和全国帆船帆板锦标赛。汕尾渔歌入选第四批国家级非遗名录。扶贫开发“双到”工作成效明显，全市164个贫困村共投入扶持资金8.15亿元，实施村帮扶项目3206个。

　　统计、审计、外事、侨务、港澳台、民族宗教、档案地方志、三防、气象、消防、防震、红十字、老年人、残疾人等工作取得新进展。

　　过去一年，我们坚持加强政府自身建设，不断完善决策机制，提高依法行政水平。坚持向人大及其常委会报告工作，向人民政协通报情况，自觉接受监督，办理省、市人大代表议案建议12件、政协提案101件。认真听取各民主党派和工会、共青团、妇联等群团意见，凝聚各方智慧，改进政府工作。大力争取上级支持，省在财政专项补助资金、深汕特别合作区及各县（市、区）产业园区基础设施建设和革命老区政策等方面，进一步加大对我市的政策倾斜和资金扶持。扎实开展党的群众路线教育实践活动，严格落实中央“八项规定”及省实施办法，深入推进政务整治和财政供养人员“吃空饷”专项清理等行动，认真查摆“四风”等方面突出问题，制订落实整改措施，建立健全长效机制，取得明显成效。市一级精简文件27.7%、会议12.8%，市、县两级考核检查活动精简90%、压缩“三公”经费39.6%，清理“吃空饷”人员1314人，工作效率和服务水平得到提高。

　　各位代表，过去一年的工作成绩来之不易，这是全市人民在省委、省政府和市委领导下，开拓创新、团结奋斗的结果，是社会各界通力合作、大力支持的结果。在此，我代表市人民政府，向广大市民和各界人士表示崇高的敬意！向长期关心支持我市经济社会发展的港澳台同胞、海外侨胞表示衷心的感谢！

　　我们也清醒地认识到，我市经济社会发展和政府工作仍存在一些困难和问题。主要表现在：一是实体经济十分薄弱。“四上”企业只有439家，居全省末位，产值超10亿元以上工业企业只有17家，销售额超亿元以上批发零售业企业仅17家，龙头企业数量偏少，单体规模不大，辐射带动能力较弱。二是财税收入问题突出。受土地相关税收收入大幅下降、汕尾红海湾电厂抵扣增值税等因素影响，全年公共财政预算收入仅增长2.2%，市直和城区地方可支配财力锐减，收支矛盾突出。财政收入质量较差，非税收入占比达53%，财税收入增长缺乏稳定性。三是社会建设与管理任务艰巨。教育创强、医疗卫生、就业服务、社会保障等民生事业有待加强，市体育中心、文化艺术馆等公共文化设施建设进展不快，资源环境、人口计生、殡葬改革、社会治安、城市管理等存在薄弱环节，陆丰“三甲”地区制贩毒形势依然严峻，社会维稳压力较大。四是行政效能建设亟需加强。有的部门协调与沟通不畅，主动服务与配合不足；有的干部发展意识不够强烈，执行力较差，工作效率不高，影响工作正常推进。上述存在的困难和问题，我们必须高度重视，采取应对措施，努力加以克服和解决。

2015年工作安排

　　2015年是全面深化改革的关键一年，是全面落实依法治国方略的开局之年，也是全面完成“十二五”规划的收官之年。随着中央依法治国战略部署的推进，省委、省政府促进粤东西北地区振兴发展战略的深入实施，深圳对口帮扶汕尾的全面深化，深莞惠经济圈各市的深度合作，加上近两年来我市在新区和园区建设、产业项目引进、投融资平台搭建、土地资源整合等方面做了大量基础工作，干部队伍干事创业热情得到激发，我市振兴发展将迎来更好的发展契机。我们必须坚定信心，抢抓机遇，全力推进全市经济社会加快发展。

　　2015年政府工作的总体思路是：全面贯彻党的十八届三中、四中全会、中央经济工作会议和省委十一届四次全会精神，按照市委六届四次全会的部署，牢牢把握加快发展这一主线，以提高经济发展质量和效益为中心，主动适应经济发展新常态，保持奋发有为的精神状态，坚持全面“融珠”，坚持实业兴市，坚持深化改革，坚持依法行政，加强民生保障，狠抓工作落实，促进全市经济社会持续健康发展，为把我市建设成为全省滨海旅游集聚区、宜居宜业宜游现代化滨海城市、珠三角产业拓展集聚地而努力奋斗。

　　2015年的主要预期目标是：地区生产总值增长10.5%；人均GDP增长9.5%；规模以上工业增加值增长18%；固定资产投资增长21%；社会消费品零售总额增长11%；外贸进出口总额增长9.5%；一般公共预算收入增长8%；城乡居民收入增长8%；居民消费价格涨幅控制在3%左右；城镇登记失业率控制在3.0%以内；节能减排约束性指标完成省下达的年度计划。

　　（一）着力推进“三大抓手”，打造发展新引擎

　　加快交通基础设施建设。加快潮惠高速公路汕尾段建设，确保陆河“县县通”路段建成通车。抓紧推进深汕高速公路长沙湾全互通出入口及潭西出入口工程。新（改）建国、省道及县乡公路250公里。加快广汕高铁、兴宁至汕尾高速公路、珠东沿海快速通道等前期工作，确保兴宁至汕尾高速公路动工建设，力争广汕高铁早日开工。规划建设市客运枢纽中心。加强与珠三角的融合，加快与深圳盐田港合作共建国际物流港口，大力推进红海湾新港区货运码头、陆丰核电厂重件码头、陆丰甲湖湾电厂配套码头工程建设。

　　加快产业园区扩能增效。坚持分类指导，分步推进，构建主业突出、功能合理的产业园区体系，确保全年新动工项目100个、力争达到120个。趁热打铁推进深汕特别合作区加快发展，主动作为，在管理授权、土地规划、征地拆迁、社会管理等方面给予深汕特别合作区更大支持，积极同深圳市一起制订实施三年行动计划，强力推进投融资、招商引资、项目用地和基础设施建设，力争开发建设5平方公里以上，完成投资100亿元，新动工项目50个，竣工投产项目10个。遵循市场经济规律，落实目标管理责任制，全力加快推进各县（市、区）园区管理机制、土地征储、项目引进、基础设施、投融资平台等工作，力争各园区开发建设1平方公里以上，其中，海丰、陆丰、红草园区新动工项目各20个，投资额各12亿元以上；陆河、红海湾园区新动工项目各10个，投资额各6亿元以上。

　　加快新区建设和城区扩容提质。坚持产城融合，统筹加快起步区建设,抓好征地拆迁和土地储备，新增征地面积4000亩。发挥振兴发展股权基金引导作用，落实基金融资，力争完成基金投资7亿元。加快招商引资及项目落地，加紧推进火车站片区、品清湖片区城市配套建设，推进工业大道西段、金鹏路等市政道路建设，督促支持城区推进红海西路工程项目，打通市区一批断头路。加快东区污水处理厂集污管网等项目建设，确保汕尾市生活垃圾无害化处理中心首期工程建成投用。完善城市综合管理与行政执法改革，以法制理念强力治理城市“脏乱差”，深入开展交通秩序、环境卫生和乱搭建、占道经营等专项整治，较大幅度改善市容市貌。积极推进县城、中心镇以及专业镇优化发展，加快完善城镇道路、公共交通、环境卫生、污水和垃圾处理等基础设施，提高城镇人口和产业集聚度。

　　（二）着力发展实体经济，推动产业优化升级

　　加快重点项目建设。全年安排市重点项目61项，年度投资173.4亿元，其中能源建设投资66.7亿元。确保陆丰核电、陆丰宝丽华甲湖湾电厂获得核准并动工建设。推动汕尾电厂5、6号机组、华润海丰电厂3、4号机组等能源项目上马建设。加快华润海丰电厂1、2号机组、华能陆丰风电、腾讯云计算中心等项目建成投产，推动胜大光电、比德能源二期等工业项目建设。抓紧建设马宫、东涌等一批输变电工程, 保障经济社会发展用电需求。安排财政资金1500万元，做好重大项目储备和前期工作。

　　做大做强实体经济。坚持实业兴市，主动承接珠三角产业转移辐射，大力发展先进制造业、高新技术产业，加快培育新能源、新材料、海洋生物、新一代信息技术等新兴产业。安排促进产业发展专项资金1000万元。落实培育大型骨干企业和扶持中小微企业发展政策措施，帮助企业解决用工、用地、融资等难题，进一步清理规范涉企行政事业性收费，加快完善中小微企业综合服务平台，积极推进企业“个转企”、“小升规”，培育壮大市场主体。

　　大力发展现代服务业。加强政策引导和新业态培育，大力发展金融服务、现代物流、文化创意等生产性服务业，加快粤东水产商贸中心等大型物流中心建设，推进广晟南储冷链物流园、广物汽车城等项目建设，打造高端商贸综合体。大力培育金融产业，推动境内外金融机构设立分支机构。加快汕尾特色产品电子商务平台建设，加强与阿里巴巴集团和深圳电商协会合作，建立县级运营中心和村级服务站，推动电子商务发展。加强信息化基础设施建设，继续推进光纤到户工程，加快4G技术应用和发展。实施快递下乡工程，推进“万村千乡”市场改造升级、农超对接，培育农村流通合作服务组织。

　　加快发展休闲旅游产业。出台加快旅游业发展意见,整合滨海山水、历史人文和运动休闲资源，促进旅游业提质升级，着力打响汕尾旅游品牌。加大宣传推介和招商引资力度，加快红海湾、金町湾、品清湖、鲘门以及赤石明热温泉、碣石玄武山、海丰湿地鸟岛等旅游区建设，打造旅游新亮点。

　　强化科技创新驱动。科学规划，分步实施，努力把深汕特别合作区建设成为国家级创新示范区。办好汕尾高新技术产业开发区。继续加强海洋产业研究院、检验检疫技术服务平台、专业镇公共技术服务平台等研发机构和创新平台建设，为主导产业转型升级提供有效的创新支撑。建立健全技术改造信息平台和项目库，大力争取省新一轮技术改造专项资金，争取2015-2017年三年全市50%以上的工业企业实施技术改造。深入实施质量强市和品牌带动战略，争取新创驰名商标、著名商标和省级以上名牌产品有新的突破。

　　（三）着力深化改革创新，激发发展活力

　　深化行政审批制度改革。继续清理精简行政审批事项，取消非行政许可审批事项。全面推进清理行政职权和编制权责清单工作。推进行政审批标准化建设，优化行政审批流程，探索建立“办事工作日”、“告知承诺”机制，公布实施行政审批标准化办事指南和业务手册。继续完善全市网上办事大厅建设，开通镇街网上办事站，加快推进政府服务事项网上全流程办理。

　　深入推进商事制度改革。继续推进“先照后证”改革，加强工商登记审批事项目录的动态管理，实现与国家目录有效对接。推进电子营业执照系统建设，打造便捷高效的网上登记注册大厅。加快市场主体信用信息平台建设，稳步推进工商营业执照、组织机构代码证、税务登记证“三证合一”登记制度改革。加快完善商事登记后续监管措施，建立健全市场主体自主负责、社会自律、群众监督、政府强化监管和依法治理的综合监管体系。

　　进一步加强投融资体制改革。认真贯彻国务院《关于创新重点领域投融资机制鼓励社会投资的指导意见》，充分发挥振兴基金及投融资平台的杠杆作用，大力推动企业发债、PPP等融资模式，鼓励引导各类资本参与城市公共基础设施和社会事业建设。加强担保平台建设，积极稳妥推进风险投资、知识产权质押融资等科技金融工作。鼓励支持企业上市，引入和推动私募基金、债券、股权融资等投融资模式。

　　加快国有企业改革步伐。深入实施国有企业股权多元化改革，探索发展混合所有制经济，推动央企、省企与我市国有企业、民营企业合作重组。继续抓好市属医疗机构、汽运总公司与央企、省企的资本合作，实现公有制经济和非公有制经济融合互补、互促共赢。

　　扎实推进其他领域改革工作。落实好中央和省其他改革任务，继续深化价格、土地、户籍等改革工作，稳步实施基层社会管理体制改革，创新村居基层治理模式。继续抓好海丰省供销综合改革县级试点工作，积极争取中小城市综合改革试点等更多项目落户我市。按上级部署要求，扎实推进全市公务用车制度改革。创新体制机制，积极探索推进华侨管理区等地区行政管理体制改革，加快建立完善深汕特别合作区、汕尾新区起步区和各产业园区管理体制，提高行政效能和服务质量。

　　（四）着力加快融入珠三角，扩大开放合作

　　强化规划的引领作用。站在珠三角的全局谋划汕尾的发展，认真对标《珠三角地区改革发展规划纲要》、《深莞惠区域协调发展总体规划》等，结合汕尾实际，精心编制《汕尾市国民经济和社会发展第十三个五年规划纲要》，修编完善汕尾城市、产业、交通、土地、环境等发展规划，为全面“融珠”提供科学指导和坚实支撑。

　　积极参与深莞惠经济圈建设。深入研究深莞惠经济圈（3+2）合作机制，借鉴深莞惠成功经验为我所用，按照“政府推动、市场驱动”的原则，积极参与深莞惠区域协作发展，进一步深化产业、环保、交通等具体项目的合作对接，助推汕尾加快振兴发展。

　　深入对接深圳对口帮扶。切实增强主体意识和主动意识，在更多领域争取深圳的帮扶，制定年度帮扶目标任务，落实帮扶项目,推动对口帮扶纵深发展和帮扶任务的落实。继续完善合作共建运营机制，深化规划、项目、管理、服务“四个对接”，构建特色产业平台，打造园区共建的示范标杆。

　　扩大外向经济发展。积极推动汕尾成为21世纪海上丝绸之路重要节点城市。充分利用深圳招商平台，积极开展定向招商、专业化招商、产业链招商、园区特色招商，支持企业“走出去”开展境外投资合作。鼓励开展跨境电子商务、零售出口，推动进口与出口、内贸与外贸互促共进。推进口岸设施建设和通关便利化，确保汕尾港红海湾港区口岸综合查验中心投入使用，海丰小漠港区实现口岸临时通关，启动陆丰甲湖港区对外开放申报工作。

　　（五）着力推进财税工作，提高经济发展质量

　　加强财税征收管理。坚持依法征税，进一步完善税源监控、税收分析、纳税评估、税务稽查“四位一体”互动机制，深入开展税源排查，抓好主体税种和重点税源征管，确保税收可持续增长。认真抓好“免抵调”及地方税种的征收管理，确保应收尽收。挖掘税收存量，规范非税收入，降低非税收入比例，着力提高财政收入质量。

　　深化财税体制改革。深化预算体制改革，建立跨年度预算平衡机制。深化民生财政保障制度改革，加大财政保障基本公共服务投入。进一步推进绩效评价改革和公务卡结算改革，稳步推进营改增改革。深化税收征管改革，推动综合治税平台建设。

　　提升依法理财水平。统筹政府性资产，整合盘活国有资产、各类闲置资金等存量资产，规范国有资源（资产）收入管理，集聚政府财力。统筹税收与非税收入，加快土地储备和拍卖出让进度，在土地经营、房地产等方面生财聚财，以土地存量的盘活提高经济增量。统筹财政资金与其他社会资金，把政府性收支全部纳入预算统筹安排，加大公共财政、政府性基金等各类资金的统筹使用，提高资金使用效率与效用；采取PPP、企业债券、股权基金等融资方式，撬动更多的社会资本支持实体经济。

　　（六）着力抓好“三农”工作，推动农村农业现代化

　　加快农业农村基础建设。建设高标准农田16万亩。抓好中小河流治理工程、小型病险水库除险加固，加快推进治洪治涝保安工程，实施小型农田水利重点县、农村水电增效扩容改造工程等项目建设。启动螺河堤围、龙潭灌区加固改造工程，加快公平灌区节水改造工程，推进三甲海堤加固达标工程、遮浪渔港一期工程。推进农村连片环境综合整治，完善农村生活垃圾收集、转运、处理体系，加大“村村通”自来水工作力度。抓好陆河省级新农村示范片建设，带动幸福村居创建和名镇名村示范村建设，改善农村人居环境，建设美丽乡村。

　　加快发展现代农业。深入开展粮食稳定增产行动，抓好“菜篮子”工程。加强现代农业产业体系建设，培育2家省级和10家市级农业龙头企业、100个农民专业合作社，积极引导农民专业合作社有效运作。推广海丰成功经验，大力引进和培育现代农业龙头企业，加快现代农业示范园区发展。大力发展海洋经济，加快渔船更新改造，推进马宫、碣石、遮浪等现代化渔港建设，发展深外海捕捞和水产品深加工业，加快建设汕尾（马宫）海洋科技产业园区。

　　深化农村综合改革。抓好陆河县农村土地承包经营权确权登记颁证试点，全面铺开农村土地承包经营权确权登记颁证工作，积极引导农村土地承包经营权有序流转。深化集体林权制度改革。全面完成农村集体资金、资产、资源管理服务平台建设，推进农村“三资”管理制度化、规范化。完善农村金融信用体系，以陆河县为试点，全面铺开农村普惠金融工作。继续推进海丰、陆河农村综合改革。

　　（七）着力抓好生态文明和社会建设，不断改善发展环境

　　加强生态建设和保护。深入实施南粤水更清行动计划，加强公平水库、赤沙水库、螺河等重要水资源保护。确保甲子、碣石污水处理厂近期建成投入使用，加快可塘、螺溪等其他5个中心镇污水处理厂建设。认真落实主要污染物总量减排考核工作，加强环境监管力度和环保能力建设，完成淘汰“黄标车”、老旧车3028辆任务。实施大气污染防治行动计划，加大固体废物、重金属污染防治力度，确保全市环境质量达标。

　　继续开展绿化大行动。做好生态控制线、林业生态红线的划定工作。继续推进林业四大重点生态工程建设，完成20万亩造林工程建设任务，建成250个村头公园。加强森林、景观林抚育，落实森林防火责任制，加强森林防火队伍和护林员建设，提高森林管护水平。

　　加强资源节约利用。加快《汕尾市海岸保护与利用规划》编制报批，加强海洋自然保护区、海洋生态文明示范区建设和管理，争取品清湖列入省美丽海湾建设试点。严守耕地红线，实施最严格节约集约用地管理，强化土地执法，抓好闲置土地清理处置。创新土地征收储备机制。加快“三旧”改造工作，推进城中村改造。

　　加大打击制贩毒品力度。深入落实《关于加强禁毒工作的实施意见》，理顺禁毒工作机制，建立禁毒工作责任追究制度。继续保持高压态势，严厉打击制贩毒犯罪活动，突出抓好清查清理、易制毒化学品管控、案件侦破、严惩“保护伞”等工作，全力缉捕负案在逃人员，积极追缴毒犯非法所得。加大禁毒宣传教育力度，继续深化“无毒社区”创建工作，营造全民参与禁毒氛围。

　　全面加强和创新社会管理。推进平安汕尾建设，加快完善立体化治安防控体系，深入开展涉黑、“两抢一盗”和涉枪、涉车等专项打击行动。加强反走私工作，大力打击非法加工经营旧服装和走私成品油活动。严格执行计划生育政策，强化计划生育服务和管理，努力稳定低生育水平。加大殡葬管理力度，进一步巩固殡改工作成果。严格落实安全生产责任制，强化监管执法、检查督查，坚决遏制重特大事故发生。切实强化食品药品质量安全管理，确保群众“舌尖”上的安全。健全应急救援体系，加强防灾减灾体系建设。完善综治信访维稳体系，及时处理群众合理诉求，维护社会和谐稳定。

　　（八）着力保障和改善民生，加快发展社会各项事业

　　强化社会保障工作。以创业带动就业，促进大学生等重点群体就业，全年城镇新增就业4.7万人，失业人员再就业1.9万人。深化与深莞惠经济圈劳务合作，大力推进劳动力技能晋升培训转移就业，全年转移农村劳动力3.7万人，参加技能晋升培训劳动力2万人次。推进城乡养老保险制度建设，加强社会保险基金监管，进一步推进异地就医即时结算，加快实现社保卡“一卡通”。提高城乡低保标准和补助水平，实现医疗救助“一站式”即时结算服务体系全覆盖。

　　大力发展教育事业。继续推进教育创强，确保年底申报教育强市督导验收。完善教育信息化建设，大力推进网络进课室。创新办学模式，加快发展学前教育、职业教育、特殊教育，支持鼓励民办教育发展。深入实施“强师工程”，加强教师队伍建设。新建1000套中小学教师公租房，加快改善教师工作和生活条件。

　　推进文化卫生事业发展。大力培育和弘扬“包容守信、务实争先”的城市精神，深入开展省文明城市、省卫生城市“双创建”工作，力争取得“广东省创建文明城市工作先进城市”称号。加快推进市体育中心、文化艺术馆等公益性基础设施建设。广泛开展全民健身活动，备战第14届省运会。加快市级医疗机构建设，推进县级公立医院改革,全面推进平价医院、平价诊室建设。继续做好农村妇女“两癌”免费筛查工作。加强H7N9流感疫情防控，保障公共卫生安全。继续做好“双拥”工作，努力创建全国和全省双拥模范城（县）。

　　全力完成扶贫工作任务。进一步增强做好新一轮扶贫开发“双到”工作的紧迫感和责任感，突出产业扶贫，确保今年全市164个贫困村集体经济收入均达到5万元以上，贫困户人均纯收入和贫困村农村人均收入分别达到全省农民人均纯收入的45%和60%以上，基本完成省扶贫任务。继续争取国家把我市纳入欠发达革命老区支持范围，享受参照中央苏区优惠政策。

　　同时，注重解决民生突出问题，继续办好“十件实事”。

　　（九）着力推进政府自身建设，不断提高依法行政水平

　　加强民主法治建设。认真落实人大及其常委会各项决议决定，坚持向人大及其常委会报告工作，认真听取各民主党派、工商联、无党派人士和各人民团体的意见建议，切实做好人大代表议案和政协提案办理工作，自觉接受各方监督。健全依法科学民主行政决策机制，对事关发展全局、群众切身利益的重大决策、重大项目，落实公众参与、专家论证、风险评估、集体讨论决定等法定程序,探索建立重大行政决策终身责任追究和责任倒查制度。抓好陆河行政复议委员会试点工作，加强应诉能力建设，建设法制型行政机关。

　　提高依法行政水平。把法治精神融入到行政管理的各个环节，深化行政执法体制改革，依法履行政府职能，规范公正文明执法。积极推行政府法律顾问制度，完善市政府常务会议学法等制度，加强公务员教育管理，运用法治思维和法治方式推动工作，提升政府工作法治化水平。认真落实行政执法责任制和责任追究制，防止行政不作为和乱作为，建设责任型行政机关。深入开展“六五”普法等活动，增强全民法治观念，促进良好社会秩序的形成。

　　强化对行政权力监督制约。认真落实党风建设责任制，完善惩治和预防腐败体系，加快构建监察、司法、审计联动的行政权力监督机制,从源头上预防和治理腐败。加快推行负面清单、权力清单、监管清单，规范权力运用。创新政务公开工作，完善基层和各领域办事公开制度，健全法律监督、民主监督和舆论监督机制，建设廉洁型行政机关。

　　加强政风行风建设。落实中央“八项规定”和“三严三实”的要求，严守政治纪律，巩固和拓展群众路线教育实践活动成果，推进作风建设制度化、常态化。全面深化政务整治行动，继续改进文风会风，大力整治“中梗阻”、“庸懒散”等问题，建设服务型行政机关。

　　提高政府执行力。按照省委省政府确定今年为“抓落实年”和市委六届四次全会的部署要求，强化行政执行力建设，聚精会神脚踏实地抓好落实，做到重实干、办实事、求实效，大兴求真务实之风，建设高效型行政机关。坚持抓早抓主动，从第一季度就狠抓工作落实，对“三大抓手”、实体经济、改革和“融珠”、社会建设和社会管理等工作实行“项目清单”制度，把每一件工作都当作一个项目来推进，细化分解任务，明确具体责任，实行定期督查，推动工作落实，坚决兑现奖惩，确保全年目标任务顺利完成。

　　各位代表，时代赋予我们重任，人民寄予我们厚望。让我们紧密团结在以习近平同志为核心的党中央周围，在中共汕尾市委的坚强领导下，在人大、政协的监督支持下，紧紧依靠全市人民，凝聚加快发展的强大合力，团结拼搏，开拓创新，务实进取，为加快汕尾振兴发展而努力奋斗!
